# Substance use disorders in bipolar patients with a painful expression

**DOI:** 10.1192/j.eurpsy.2023.470

**Published:** 2023-07-19

**Authors:** J. Chabert, R. Icick, J. Cabé, M.-C. Patoz, X. Moisset, O. Godin, S. Gard, J. Loftus, V. Aubin, R. Belzeaux, C. Dubertret, Y. Lestrat, N. Mazer, A. De Premorel, P. Roux, M. Polosan, T. Schwitzer, B. Aouizerate, B. Isabelle, B. Etain, R. Moirand, E. Olié, E. Haffen, M. Leboyer, P. Courtet, P.-M. Llorca, G. Brousse, L. Samalin

**Affiliations:** ^1^CHU Clermont-Ferrand, Université Clermont Auvergne, Institut Pascal, Clermont Ferrand; ^2^CHU Lariboisière-Fernand Widal, INSERM UMRS 1144, Université de Paris Cité, Paris; ^3^CHU Clermont-Ferrand, University of Clermont Auvergne, Institut Pascal; ^4^CHU Clermont-Ferrand, Université Clermont Auvergne, Institut Pascal, Clermont-Ferrand; ^5^Fondamental Fondation, Créteil; ^6^Hospitalier Charles Perrens, INRAE UMR 1286, University of Bordeaux, Bordeaux, France; ^7^Center Hospitalier Princesse Grace, Monaco; ^8^Centre Hospitalier Princesse Grace, Monaco; ^9^AP-HM, INT-UMR7289, Aix-Marseille Université, Marseille, France; ^10^Université de Paris, INSERM UMR1266, Hôpital Louis Mourier, Colombes; ^11^Equipe DisAP-PsyDev, Université Versailles Saint-Quentin-en-Yvelines - Paris-Saclay, Villejuif; ^12^Université Grenoble Alpes, CHU Grenoble, Grenoble Institut des Neurosciences (GIN) Inserm U 1216, Grenoble; ^13^Université de Lorraine, Inserm U 1254, CHU Nancy, Laxou; ^14^Hospitalier Charles Perrens, INRAE UMR 1286, Université de Bordeaux, Bordeaux; ^15^CHU Lariboisière-Fernand Widal, Paris; ^16^INSERM U1028; CNRS UMR5292; University Lyon 1, Villeurbanne,. ΨR2 Team, CH Le Vinatier, Bron; ^17^Lapeyronie CHU Montpellier, Institut de Génomique Fonctionnelle, Université de Montpellier, Montpellier; ^18^CIC-1431 INSERM, CHU de Besançon, Besançon; ^19^CHU Henri Mondor, Université Paris Est Créteil, INSERM U955, Créteil; ^20^Lapeyronie CHU Montpellier, Montpellier, France

## Abstract

**Introduction:**

Bipolar Disorder (BD) is a common psychiatric disease. It has been demonstrated a long time ago that bipolar patients are more painful than the healthy subjects. Substance use disorder is a frequent comorbidity in BD, but also in painful patients. The aim of our study was to analyze if bipolar patients with a painful expression have more substance use disorder than bipolar patients without pain.

**Objectives:**

The aim of our study was to analyze if bipolar patients with a painful expression have more substance use disorder than bipolar patients without pain

**Methods:**

We included all bipolar patients from the FACE-BD cohort which is a prospective cohort of French outpatients with BD enrolled at the 12 advanced Centers of Expertise in Bipolar Disorder (CEBD). Pain has been evaluated by the “pain item” of the EQ-5D scale and we divided subjects in four categories: “no pain”, “slight pain”, “moderate pain”, “severe or extreme pain”. A multivariate analysis was performed to identify differences between each pain’s groups according to the kind of substance use disorder, psychiatric comorbidities and clinicals data.

**Results:**

The cohort enrolled 1897 bipolar patients, 970 had no pain (51.1%), 507 had slight pain (26.7%), 298 had moderate pain (15.7%) and 122 had severe or extreme pain (6.4%). We found significant differences according to age, comorbidities and clinicals data with older, more anxious, and more severe patients more represented in the more painful groups. Painful bipolar patients had also more frequently lifetime substance use disorders (alcohol, opioid, sedative, marijuana) and we were able to characterize different profiles in bipolar patients.

**Conclusions:**

Bipolar patients with a painful expression had more risks to have a lifetime substance use disorder, an anxiety disorder, and a higher score on MADRS. Interestingly, subjects seemed to prefer substances with anxiolytic or antalgic effects during the acute intoxication as alcohol, marijuana, opioid and sedatives.

**Disclosure of Interest:**

None Declared

